# High-content screening (HCS) workflows for FAIR image data management with OMERO

**DOI:** 10.1038/s41598-025-00720-0

**Published:** 2025-05-09

**Authors:** Riccardo Massei, Wibke Busch, Beatriz Serrano-Solano, Matthias Bernt, Stefan Scholz, Elena K. Nicolay, Hannes Bohring, Jan Bumberger

**Affiliations:** 1 Research Data Management - RDM, Helmholtz Centre for Environmental Research (UFZ), Permoserstraße 15, 04318 Leipzig, Germany; 2 Department Monitoring and Exploration Technologies - Helmholtz Centre for Environmental Research (UFZ), Permoserstraße 15, 04318 Leipzig, Germany; 3 Department Ecotoxicology - Helmholtz Centre for Environmental Research (UFZ), Permoserstraße 15, 04318 Leipzig, Germany; 4 Euro-BioImaging ERIC Bio-Hub, EMBL Heidelberg, Heidelberg, Germany; 5 Department Computational Biology and Chemistry - Helmholtz Centre for Environmental Research (UFZ), Permoserstraße 15, 04318 Leipzig, Germany; 6 German Centre for Integrative Biodiversity Research (iDiv) Halle-Jena-Leipzig, Puschstraße 4, 04103 Leipzig, Germany

**Keywords:** Data processing, Data publication and archiving

## Abstract

High-content screening (HCS) for bioimaging is a powerful approach to studying biological processes, enabling the acquisition of large amounts of images from biological samples. However, it generates massive amounts of metadata, making HCS experiments a unique data management challenge. This data includes images, reagents, protocols, analytic outputs, and phenotypes, all of which must be stored, linked, and made accessible to users, scientists, collaborators, and the broader community to ensure sharable results. This study showcases different approaches using Workflow Management Systems (WMS) to create reusable semi-automatic workflows for HCS bioimaging data management, leveraging the image data management platform OMERO. The three developed workflows demonstrate the transition from a local file-based storage system to an automated and agile image data management framework. These workflows facilitate the management of large amounts of data, reduce the risk of human error, and improve the efficiency and effectiveness of image data management. We illustrate how applying WMS to HCS data management enables us to consistently transfer images across different locations in a structured and reproducible manner, reducing the risk of errors and increasing data consistency and reproducibility. Furthermore, we suggest future research direction, including developing new workflows and integrating machine learning algorithms for automated image analysis. This study provides a blueprint for developing efficient and effective image data management systems for HCS experiments and demonstrates how different WMS approaches can be applied to create reusable, semi-automated workflows for HCS bioimaging data management using OMERO.

## Introduction

**Fig. 1:**
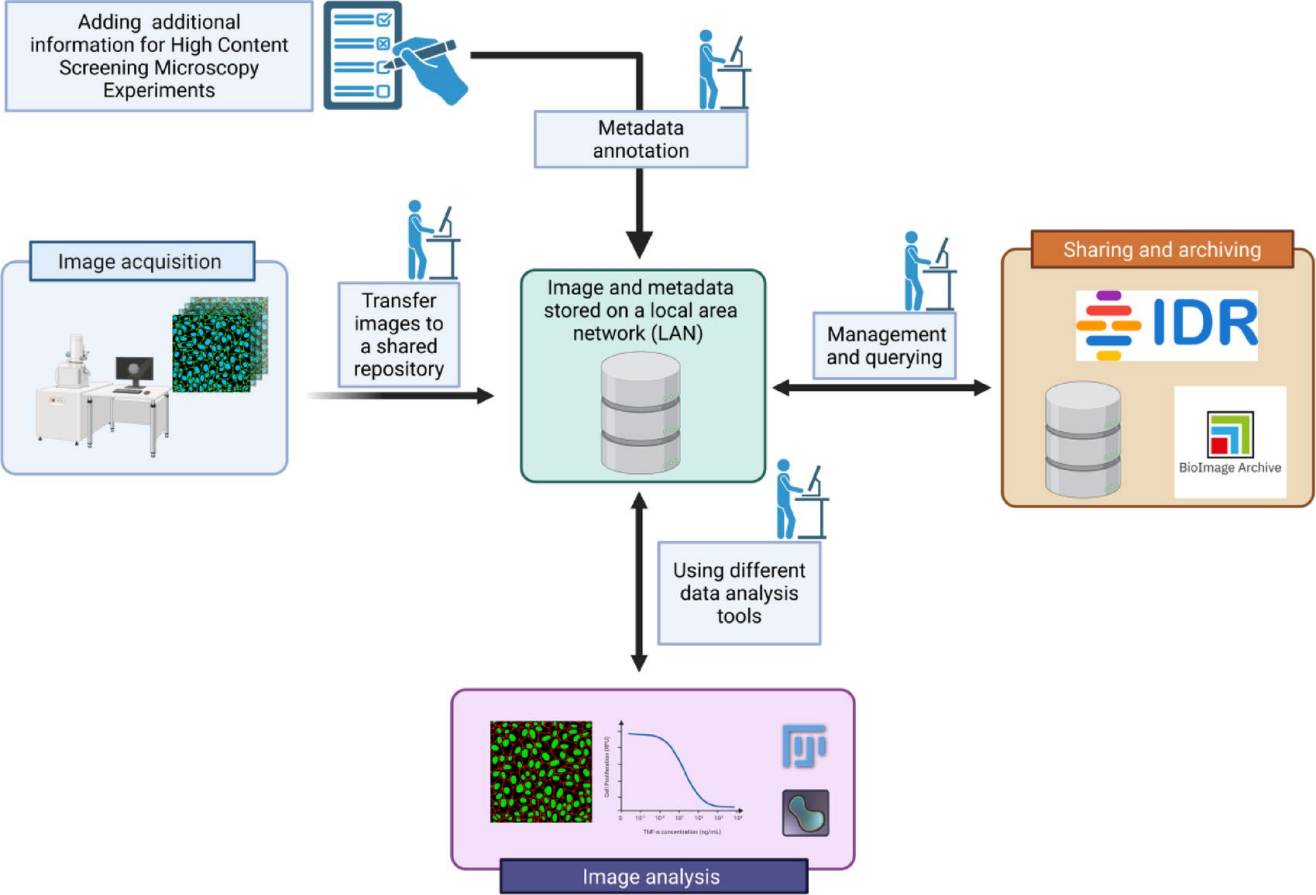
An example of a local file-based storage system for HCS bioimaging. After image acquisition, data can be transferred to a shared repository. If additional metadata is available (e.g., information on experimental conditions or the biological sample), it can be added and transferred to the image storage location. From this location, data can be fetched to perform further analysis or be shared on public repositories (e.g., Image Data Repository (IDR), BioImage Archive (BIA)). The figure was created in BioRender. Massei, R. (2025) https://BioRender.com/g74o795.

High-content screening (HCS) for bioimaging involves the automated acquisition of images from a large number of biological samples, aiming to extract both quantitative and qualitative information from these images. Since HCS experiments can produce up to a hundred thousand images from a single acquisition, a vast amount of metadata is also generated in each experiment, capturing critical information about the biological processes captured in the images and the experimental setup^1^. This makes HCS experiments a unique data management challenge, requiring integrating and linking multiple large sets of structured and unstructured data such as images, reagents, protocols, analytic output, and phenotypes^2^. All metadata must be stored, linked, and made accessible to users, scientists, collaborators, and the broader community to produce sharable results. HCS data management often relies on a file-based approach, where metadata and binary data are stored in files and folders on a local file system, typically using a hierarchical structure^2^. This approach involves manually storing data in separate files: metadata files contain descriptive information such as experimental conditions, assay settings, and sample details, while binary data files store the actual experimental results, typically in the form of images or videos. After the acquisition, data is accessed for further analysis using analysis software tools such as Fiji or Napari. Finally, data can be shared on public repositories such as the Bio Image Archive (BIA) or the Image Data Resource (IDR)^3^. An overview of this manual data management approach is shown in Fig. 1.

This manual, local file-based storage system might be prone to errors, especially when maintained without the support of a robust data management system. Furthermore, managing thousands of images produced in an HCS experiment becomes increasingly complex. A more effective solution would be implementing a dedicated platform integrating unstructured metadata with image data while supporting a well-defined, reproducible workflow for data processing, management, and storage.

Workflows are the keystone of bioimage analysis since they allow process monitoring and facilitate the integration of image processing, with metadata automatically included during image upload^4–6^. Imaging workflows can also be shared and reused among different laboratories, improving reproducibility among international institutions applying similar analysis methods^5^.

Several Workflow Management Systems (WMS)^7^, such as Galaxy, KNIME, Nextflow, or Snakemake, are already used for bioimage analysis workflows. However, their application for image data management remains underexplored and not widely adopted. An automated bioimage workflow can bridge the local system and the dedicated platform by consistently transferring images in a structured and reproducible manner across different locations. Such an approach would significantly improve efficiency, reduce the likelihood of errors, and enhance data consistency and reproducibility^6^.

This work showcases different approaches using WMS to create reusable semi-automated workflows for HCS bioimaging data management. We demonstrate how transitioning from a local file-based storage system to an automated and agile image data management framework is feasible. Specifically, we developed (1) two general workflows for automated data upload and (2) applied them to two specific biological exemplary use cases. The three developed workflows incorporate the image data management platform OMERO^8^, a powerful and flexible image data management system capable of handling large datasets, making it an ideal choice for bioimaging applications. This approach facilitates the management of large amounts of data, reduces the risk of human error, and enhances the efficiency and effectiveness of image data management.

## Methods

### An image data management framework

**Fig. 2:**
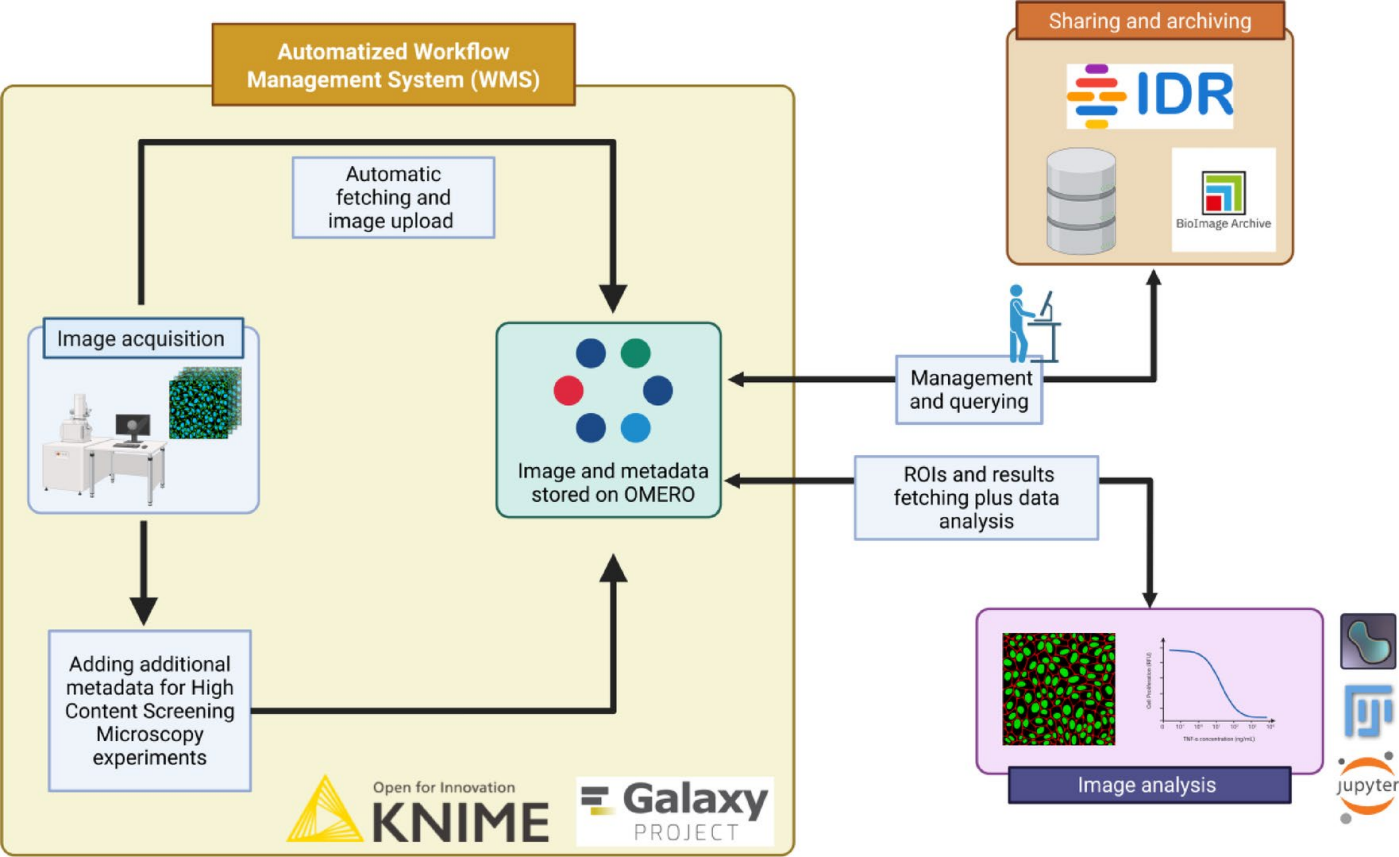
A potential automated high content screening (HCS) for image data management workflow using a dedicated workflow management system integrated with OMERO. Image was Created in BioRender. Massei, R. (2025) https://BioRender.com/k21s891.

The first step in designing a data management framework for bioimaging is to integrate a robust and reliable data management system together with a WMS (Fig. 2). This framework should include the ability to transfer images from a local storage location using a WMS to a remote data management repository. Combining these two components allows researchers to easily manage their bioimaging experiments, ensuring data accessibility, tracking, and provenance. Finally, to ensure the successful implementation and adoption of the framework, it is crucial to have a testing dataset available for testing its functionalities. This dataset should contain a diverse range of bioimaging data, including images and metadata, to evaluate the framework’s effective management of data efficiently.

### Software tools

#### OMERO—image data and metadata management system

OMERO (Open Microscopy Environment Remote Objects)^8^ is one of the most flexible and widely used open-source tools for HCS datasets and metadata management. It is an open-source software platform designed to manage, visualize, and analyze large biological image datasets. Developed by the Open Microscopy Environment (OME) consortium, OMERO provides researchers with a centralized repository to store images and metadata, tools for collaborative sharing, and advanced functionalities for image processing and analysis^8^. OMERO connects a PostgreSQL relational database, a Lucene-based search index, a filesystem-based image repository, and an HDF-based tabular data store. It supports a wide range of microscopy formats and integrates with various analytical tools, facilitating streamlined workflows in life science research. OMERO has been used in the past for the metadata management of HCS data^2^, and it allows the visualization of the data in plates and screens.

Furthermore, the Java library Bio-Formats^9^ fully integrates with the OMERO servers. Bio-Formats allows reading and writing life sciences image file formats. It is capable of parsing both pixels and metadata for a large number of formats, as well as writing to several formats. The primary goal of Bio-Formats is to facilitate the exchange of microscopy data between different software packages and organizations. It achieves this by converting proprietary microscopy data into an open standard called the OME data model, particularly into the OME-TIFF file format.

Data management with OMERO can take place using different applications such as OMERO.web and OMERO.insight. OMERO.insight is a desktop application available on different operating systems that allows users to upload images into OMERO easily. It is particularly user-friendly, enabling inexperienced users to perform routine data management tasks. OMERO.web is a framework for building web applications for OMERO. It uses Django to generate HTML and JSON from data retrieved from the OMERO server. OMERO.web acts as a Python client of the OMERO server using the OMERO API and as a web server itself. As a major example, IDR is a combination of the standard OMERO.web API with additional searching features, providing a JSON-based API for accessing image datasets, thumbnails and metadata. An overview of how to use OMERO.insight and OMERO.web is provided in a recent publication from the I3D: Bio Material^10^.

#### Galaxy and KNIME—workflow management systems (WMS)

To address the need for efficient and reusable data workflows in bioimaging, we investigated various tools that combine bioimage analysis capabilities with data transfer. We focused on the WMS Galaxy and KNIME to build our processing workflows. Galaxy is a robust and collaborative platform for data analysis, providing a user-friendly interface for processing extensive datasets. With Galaxy, users can version tools, annotate workflows, share them with collaborators, or make them public, significantly enhancing the FAIRness of data analysis workflows. Furthermore, Galaxy has a dedicated interface for image data analysis, providing a comprehensive suite of imaging tools and workflows tailored specifically for imaging scientists. Galaxy offers integration with several bioimage analysis tools, including CellProfiler and Cellpose^11^, which significantly enhances its potential for image analysis in a free, web-based cloud environment. Additionally, Galaxy also integrates with Napari and Jupyter Notebooks, further increasing its capabilities for image analysis. The KNIME (Konstanz Information Miner) is also an analytical platform that allows users to create modular pipelines. It supports over 140 image formats and provides preprocessing, segmentation, feature extraction, tracking, and classification of images. Workflow design takes place locally on the KNIME Analytical Platforms. Workflows can be saved as archive files and shared on different cloud environments, namely the KNIME Community Hub or Business Hub.

**Fig. 3:**
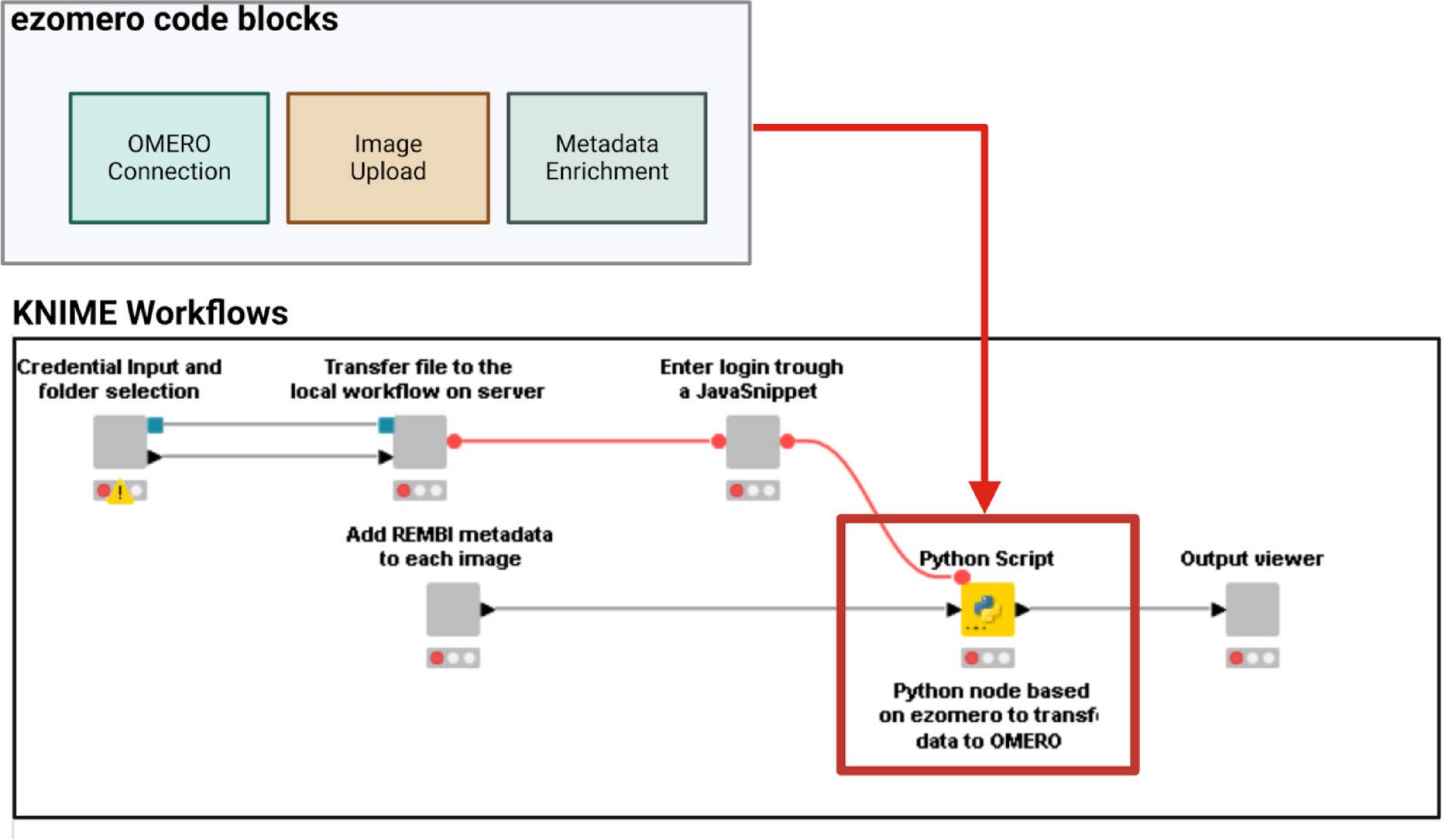
An example of a KNIME workflow for OMERO upload. Blocks of ezomero codes are inserted in the KNIME Python script nodes to perform tasks for image upload and metadata enrichment.

Galaxy and KNIME can use the OMERO Python API, allowing Python programmers to integrate their applications with OMERO for data management and transfer. In particular, ezomero^12^ is a developed Python library that provides convenient functions for interacting with the OMERO image data management system. It enables users to modify workflows, add steps during image uploads, attach files, and navigate image data using tags and Key-Value (KV) pairs. Ezomero also supports the association of Regions of Interest (ROIs) to each image in an HCS-specific fashion. To this goal, the Galaxy OMERO-suite^13^ has been developed in UFZ to simplify the transfer of data and metadata into an OMERO instance and can be integrated with image analysis workflows to manage the data. The OMERO-suite can be installed on a local Galaxy instance to perform various Research Data Management (RDM) tasks linked to OMERO. Table 1. provides an overview of the different tools of the OMERO-suite. With KNIME, the KNIME Python Integration needs to be installed on KNIME and a Conda environment with ezomero installed needs to be configured. This allows Python scripts and custom code to be included in workflows, enabling the integration of ezomero code blocks to perform OMERO-based RDM tasks (Fig. 3). This flexibility enables the creation of tailored solutions for RDM processes, including metadata enrichment and ROI uploads, which can be executed on a KNIME server, eliminating the need for local KNIME installations.

**Table 1:** List of tools from the OMERO-suite which can be used in galaxy to interact with an OMERO instance.

Class	Galaxy tool	Wrapper command function
OMERO database fetch	idr_download_by_ids	Download image data from the IDR (Image Data Resource) or from a private OMERO instance
OMERO data import	omero_import	Import images into a user-defined OMERO server. Create a new dataset or use a pre-existing one
	omero_metadata_import	Import Key-Value Pairs and Tables into a user-defined OMERO server
	omero_roi_import	Import Region Of Interest into a user-defined OMERO server
OMERO data get	omero_get_id	Get the ID of an OMERO object (i.e., images, datasets, projects, screen, plates)
	omero_get_value	Get Table, Annotation or Tags from an OMERO object (i.e., images, datasets, projects, screen, plates)
OMERO data filter	omero_filter	Filter OMERO objects by KV pairs, Tags or filename

GitHub repository: https://github.com/Helmholtz-UFZ/galaxy-tools/tree/main/tools/omero.

### Datasets

#### Zebrafish HCS dataset

The first HCS dataset (DZF) was published by^14^ and consists of zebrafish image data collected using the Vertebrate Automated Screening Technology (VAST)^15^. Images are processed further using FishInspector^16^, a software that allows users to annotate and quantify morphological structures in the zebrafish and calculate additional specimen features such as length, tail curvature and body shape. All Regions of Interest (ROIs) are saved as JSON and CSV files with coordinate data for the shapes, enabling further feature calculations. The full dataset is available on the BIA under the accession number S-BIAD954. A subset of the dataset was created and uploaded in Zenodo for testing at the link https://zenodo.org/records/14790777.

#### Cell lines HCS dataset

The second HCS dataset (DCL) includes images of cytoplasm-to-nucleus translocation of the transcription factor NFκB in MCF7 (human breast adenocarcinoma cell line) and A549 (human alveolar basal epithelial) cells in response to TNFα concentration. Images were acquired at 10x magnification using the CellCard reader from Vitra Bioscience. Each well contains one field with two images: a nuclear counterstain (DAPI) image and a signal stain (FITC) image. We used as testing dataset the image set BBBC014v1 provided by Ilya Ravkin, available from the Broad Bioimage Benchmark Collection^17^. A subset of the dataset was created and uploaded in Zenodo for testing at the link https://zenodo.org/records/14205500.

## Results

The following workflows were developed using (1) the local UFZ Galaxy instance where all the tools of OMERO-suite were installed and (2) the KNIME analytics platform 5.4. An overview of all developed workflows and their location at the WorkflowHub^18^ is available in Table 2. Workflows can only be run on Galaxy instances in which the Galaxy OMERO-suite has been previously installed.

**Table 2:** An overview of the reusable bioimaging pipelines designed for use-case-specific scenarios.

Platform	Workflow	Open Link
KNIME	General import and metadata enrichment VAST OMERO upload VAST metadata enrichment VAST ROI upload	https://workflowhub.eu/workflows/1265 https://workflowhub.eu/workflows/1257
Galaxy	General import Cell segmentation, OMERO upload, metadata enrichment and ROI upload	https://workflowhub.eu/workflows/1258 https://workflowhub.eu/workflows/1259

### Workflow 1—general import workflow for HCS bioimaging data

**Fig. 4:**
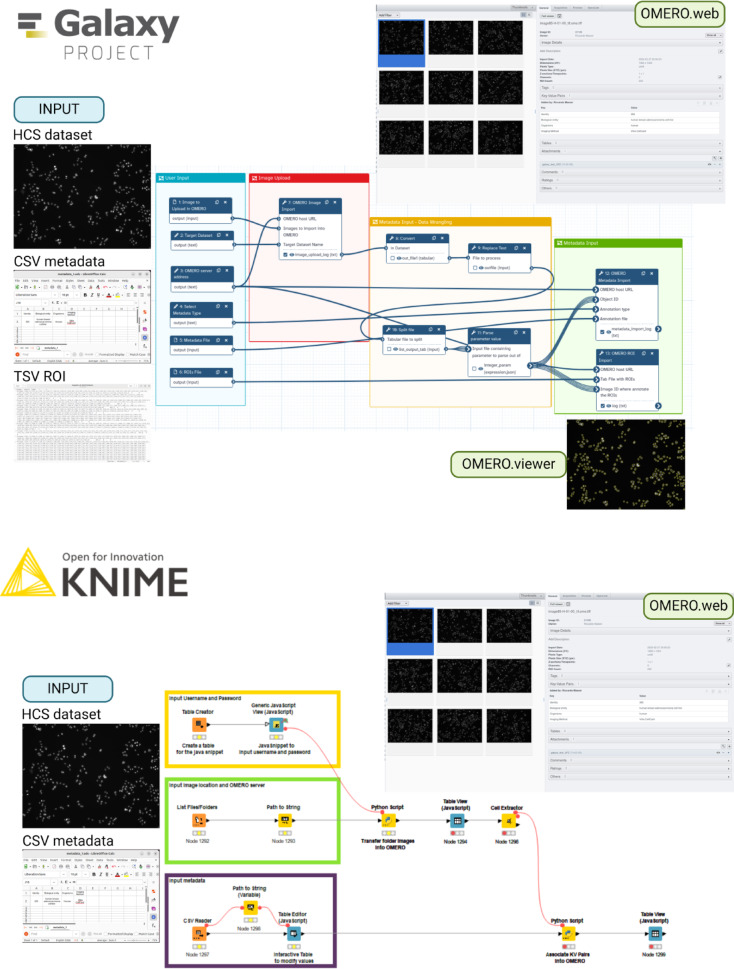
Two examples of workflows for OMERO upload using Galaxy and KNIME (Workflow 1). Testing datasets are available on the Broad Bioimage Benchmark Collection under the accession number BBBC014 (Ljosa et al.^17^). Image was created in BioRender. Massei, R. (2025) https://BioRender.com/xiy0tjb and https://BioRender.com/A69m173.

We developed a general workflow using Galaxy and KNIME to import data into OMERO (Fig. 4). The Galaxy workflow is based on three main tools from the OMERO-suite: OMERO import, OMERO metadata import, and OMERO ROI import. The workflow is based on six user inputs: the OMERO server address, the target dataset name to create in OMERO, the dataset to upload, the metadata file containing the information to upload, and another dataset containing the ROIs. Credentials for OMERO are stored in Galaxy in the user preferences.

The KNIME workflow has three main inputs from the user: (1) the user credentials to access the OMERO, input using a Java Snippet node; (2) the image location folder, and (3) the metadata input in a CSV form. All inputs are transferred to the target OMERO server using a Python node containing blocks of ezomero code. The specific workflow can be locally run on the KNIME Analytics Platform but can be easily made interactive and executed on a cloud KNIME server. It is important to mention that the credentials are visible in the Python script node and attention must be paid to keeping the credentials private.

### Workflow 2—zebrafish embryos HCS data analyzed with FishInspector

**Fig. 5:**
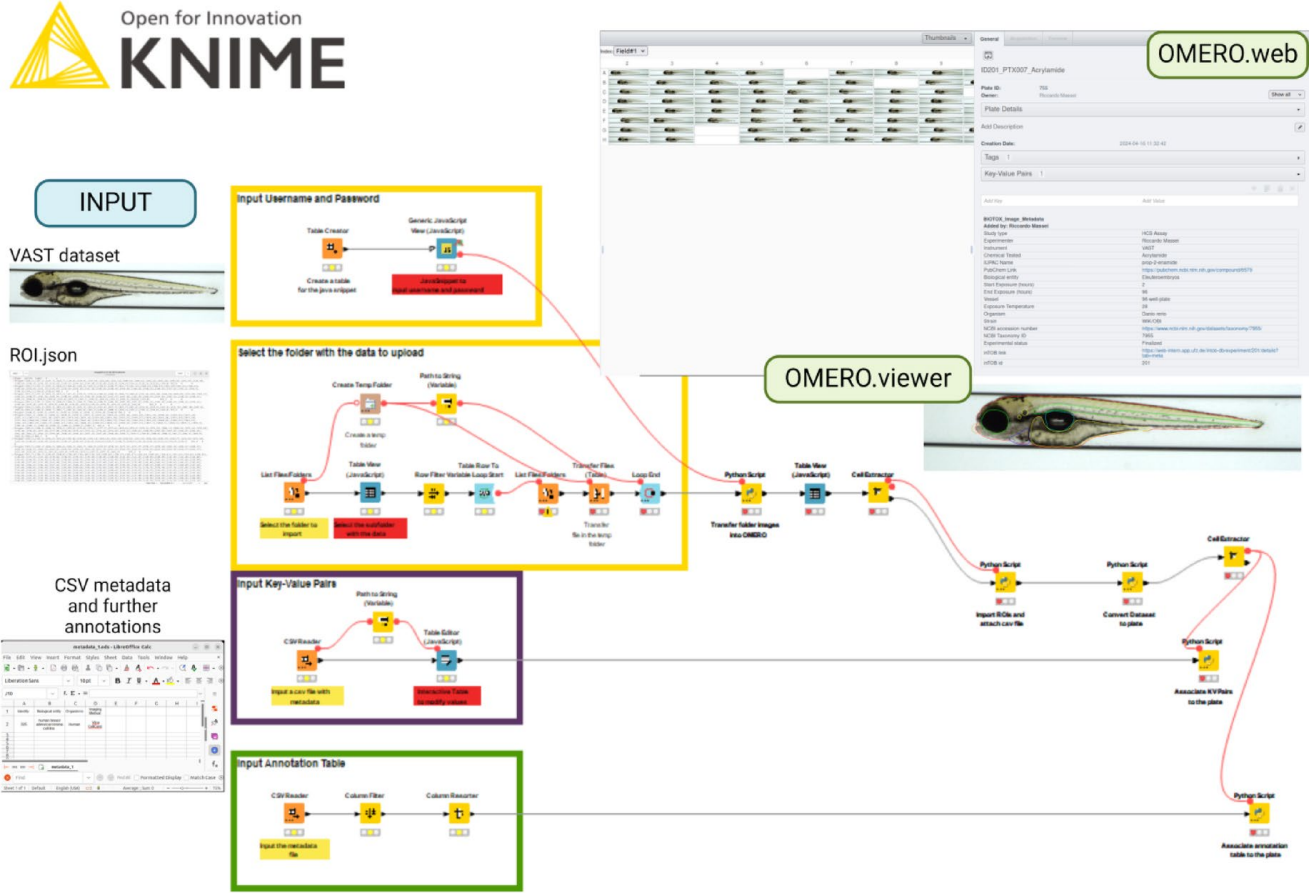
Overview on Workflow 2 Zebrafish embryos HCS data analyzed with FishInspector. Image was created in BioRender. Massei, R. (2025) https://BioRender.com/wewmfnw.

Starting from Workflow 1 build with KNIME, we created a specific workflow for the DZF, which was acquired with a VAST system using KNIME (Fig. 5). In this case, the input consisting into a folder contains the image data and the JSON file with ROIs annotation created using the software FishInspector.

We added three additional branches to Workflow 1, each adding new features. One branch was developed to parse the JSON file ROIs coordinates and format them in a readable manner by ezomero, allowing for uploading them into OMERO and visualizing them in OMERO.viewer. Additionally, this branch enables uploading the JSON file as an attachment to the image itself in a single step. A second branch was developed to automatically convert the OMERO dataset into plates for HCS visualization in OMERO. The well position is automatically read from the file name following a well identification regex which can modify according to the needs. In this way, each image is accurately mapped to its corresponding well in a 96-well plate, with multiple images collected from the same well consolidated and displayed together in that well. The third branch enables the uploading of additional results to the plate in a tabular format, which is particularly useful for linking results from external tests or analyses to the corresponding imaging experiment. This feature provides a more comprehensive understanding of the data, which can then be visualized in OMERO.table.

### Workflow 3—nuclei segmentation of cell lines in galaxy

**Fig. 6:**
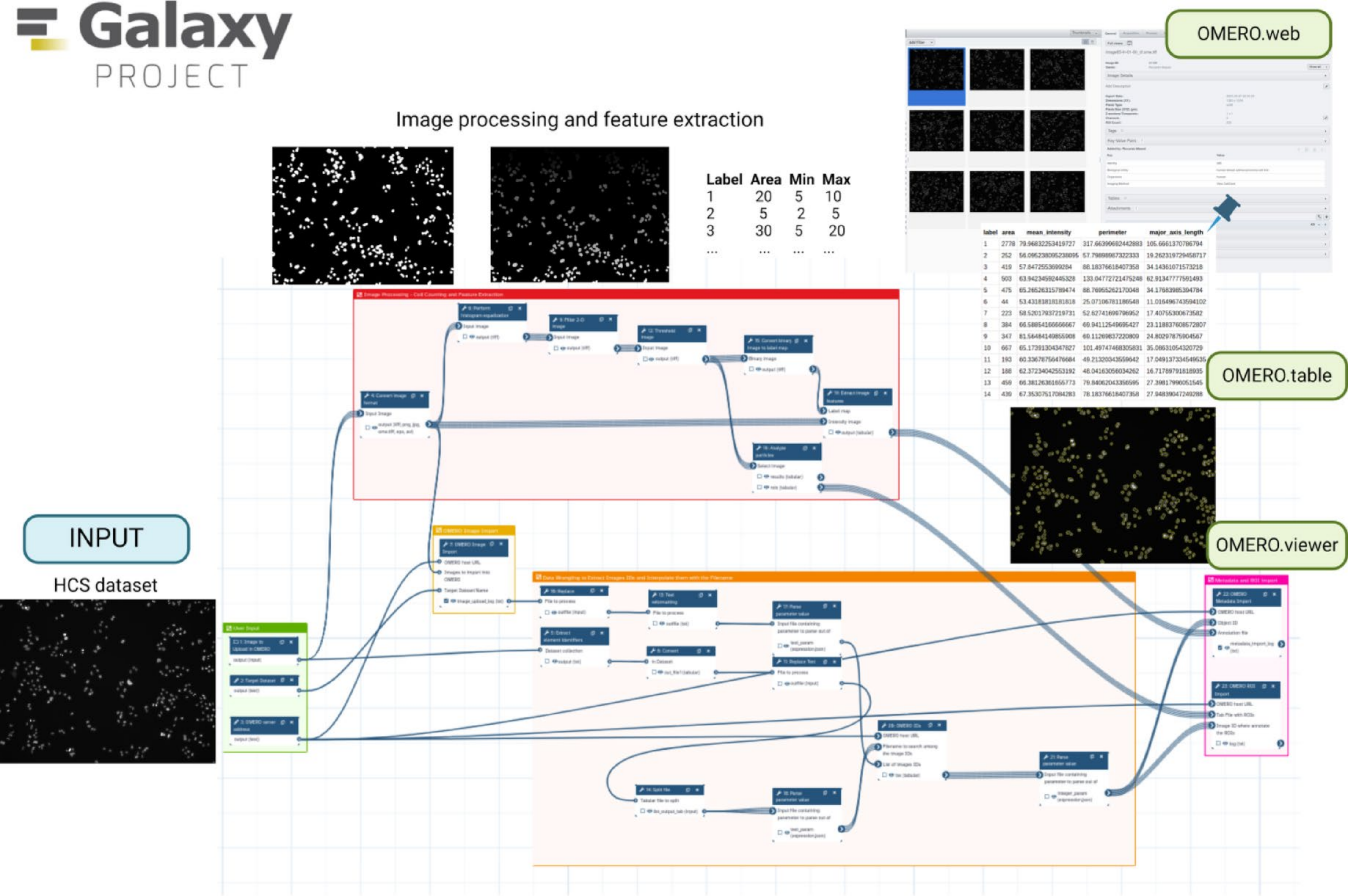
Overview on Workflow 3 Nuclei segmentation of cell lines in Galaxy. Image was created in BioRender. Massei, R. (2025) https://BioRender.com/8awy78c. A better overview of the workflow in higher resolution can be found at the WorkflowHub under the accession number 1259.

Starting from Workflow 1 build with Galaxy, we created the first integrative workflow combining image data processing together with uploading into OMERO (Fig. 6). Workflow 3 requires that the OMERO-suite is installed on the Galaxy instance.

To achieve our goal, we used the DLC dataset, which consists of 2-channel cell line image data. The first step involved converting the images to OME.TIFF format and uploading them into OMERO using the OMERO import tool. While the upload was taking place, .we used ImageJ-based Galaxy tools to perform further image pre-processing steps. This included histogram equalization, filtering to remove noise, and normalization of the images. Next, we applied Otsu’s thresholding^19^ to separate the nuclei from the background, followed by connected component analysis to further separate the detected nuclei from each other. The final result was the creation of ROIs with their corresponding coordinates, which were saved as tabular files. These features were extracted from the analysis, and all the results were associated with the target dataset, where the images had previously been uploaded. In particular, the ROIs created were associated with each image using the OMERO ROI import tool and could be visualized in OMERO.viewer. The results table with the feature extraction results could be visualized using OMERO.table. The workflow required an additional data wrangling section where image IDs were dynamically fetched from OMERO to associate the produced ROIs with the images stored in OMERO.

## Discussion and conclusion

Effective data management strategies are needed for reproducibility and long-term preservation to address the increasing volume of digital images in life sciences^20,21^. The present work demonstrated how HCS bioimaging data management processes can be automated using two different WMS, Galaxy and KNIME. The developed workflows can automate processes that would otherwise be performed manually, with the risk of errors and decreased data processing efficiency. Most importantly, the Galaxy and KNIME workflows allow users without programming skills to easily create workflows according to their own needs using reusable tools or blocks of code. This simplifies the development and implementation of complex workflows, making it easier for users to achieve their goals without requiring coding expertise. Starting from two general-purpose workflows, we showcased how they can be modified and implemented to achieve more complicated workflows. Starting from a general upload workflow, it is possible to implement modular steps to include additional data management features or even image processing features.

The integration of OMERO into workflows enhances the FAIRness^22^ of the image data management framework. OMERO enables structured storage of metadata, images, and results files, facilitating data sharing among collaborators and remote access worldwide. Notably, the Bio-Formats Galaxy tool and KNIME node simplify image format conversion before uploading to OMERO. However, while integrating file conversion within WMS offers a user-friendly interface, it can be slower, less flexible, and more resource-intensive for large datasets. The native Bio-Formats, on the other hand, provides faster and more dynamic conversions, promoting compatibility among different microscopy file formats, albeit with potential compatibility issues with older systems and more complex error handling. Both WMS can be modified to allow the direct inspection of data from OMERO as shown in Fig. 2. The current workflows can include a step for fetching data from OMERO or IDR using the “IDR/OMERO download” Galaxy tool as shown by Serrano-Solano et al.^23^ to further process it, and re-uploading the analyzed data back into OMERO. Potential workflow implementations could include new tools for directly uploading data from public sharing repositories like the BIA or IDR, automating the transfer of private data to public repositories in a reproducible manner. Many Python-based functionalities can also be encapsulated as Galaxy tools or KNIME nodes to ease data sharing. For instance, the OME team’s omero-cli-transfer could be wrapped as a Galaxy tool to facilitate data transfers between different OMERO servers and BIA using Galaxy.

When comparing the two WMS used in this study, we arrived at the same conclusion pointed out by Wollmann et al.^6^. Galaxy and KNIME can perform similar tasks in microscopy image analysis within their respective environments, they are not interchangeable and are separated by design. Galaxy users can rely on a strong community that develops new solutions and tools daily to improve workflow developments. As a major trend, research groups are shifting towards Next Generation File Formats (NGFF) for data processing and storage and analysis with the goal of standardizing multiscale and multimodal imaging data. In this context, Galaxy supports the Zarr file format and the Vizarr tool was recently integrated into Galaxy, enabling lightweight, entirely client-side rendering of multiscale Zarr images in web-based environments and interactive notebooks^24^. Additionally, Galaxy now includes an NGFF file format conversion tool that allows users to convert any file format to OME-Zarr. As more image data analysis and management tools adopt OME-Zarr, we expect broader uptake within the community.

As a prospective, further WMS can be explored for developing similar workflows in an open source environment. JIPipe and Nextflow are two examples that provide advanced solutions for bioimaging workflow automation. JIPipe is designed for researchers who need to process complex image datasets without coding expertise^25^. As for KNIME and Galaxy, it offers a node-based, visual programming environment tightly integrated with ImageJ and Fiji, allowing users to design modular workflows. JIPipe is particularly valuable for biologists and medical researchers who require automation but lack programming skills. Nextflow, on the other hand, is built for scalability and parallelization, making it well-suited for handling large-scale bioimage datasets that require extensive computational resources^26^. Based on Groovy, researchers can define workflows that can run seamlessly across local machines, high-performance computing clusters, and cloud platforms. With built-in support for containerization technologies like Docker and Singularity, Nextflow ensures that workflows remain portable and reproducible across different environments.

In conclusion, we demonstrated that WMS such as Galaxy and KNIME can significantly enhance data management automation for HCS bioimaging. Our workflows facilitate the transfer of imaging data and essential experimental metadata into OMERO. Additionally, integrating image processing into a Galaxy tool further improves the reproducibility of analyses. Collectively, these advancements enhance the FAIRness of image data management with OMERO and open new avenues for developing and applying WMS in image analysis and research data management.

## Data Availability

All workflows and further instructions and conda environment setup to run the KNIME workflows are shared on the WorkflowHub (https://workflowhub.eu/projects/293). The full zebrafish dataset is available on the BIA under the accession number S-BIAD954. A subset of the dataset was created and uploaded in Zenodo for testing at the link https://zenodo.org/records/14790777. The full human cell line dataset is available on the Broad Bioimage Benchmark Collection under the accession number BBBC014^17^. A subset of the dataset was created and uploaded in Zenodo for testing at the link https://zenodo.org/records/14205500.

## Abbreviations

API: Application Program Interface
BIA: Bio image archieve
FAIR: Findable, accessible, interoperable and reusable
HCS: High-content screening
IDR: Image data resource
JSON: JavaScript Object Notation
KV: Key-value
OME: Open microscopy environment
OMERO: Open microscopy environment remote objects
PIL: Python Imaging Library
RDM: Research data management
ROI: Region of interest
VAST: Vertebrate automated screening technology
WMS: Workflow management system
