# Correction: High-content screening (HCS) workflows for FAIR image data management with OMERO

**DOI:** 10.1038/s41598-026-44073-8

**Published:** 2026-03-16

**Authors:** Riccardo Massei, Wibke Busch, Beatriz Serrano-Solano, Matthias Bernt, Stefan Scholz, Elena K. Nicolay, Hannes Bohring, Jan Bumberger

**Affiliations:** 1 Research Data Management - RDM, Helmholtz Centre for Environmental Research (UFZ), Permoserstraße 15, 04318 Leipzig, Germany; 2 Department Monitoring and Exploration Technologies - Helmholtz Centre for Environmental Research (UFZ), Permoserstraße 15, 04318 Leipzig, Germany; 3 Department Ecotoxicology - Helmholtz Centre for Environmental Research (UFZ), Permoserstraße 15, 04318 Leipzig, Germany; 4 Euro-BioImaging ERIC Bio-Hub, EMBL Heidelberg, Heidelberg, Germany; 5 Department Computational Biology and Chemistry - Helmholtz Centre for Environmental Research (UFZ), Permoserstraße 15, 04318 Leipzig, Germany; 6 German Centre for Integrative Biodiversity Research (iDiv) Halle-Jena-Leipzig, Puschstraße 4, 04103 Leipzig, Germany

Correction to: *Scientific Reports* 10.1038/s41598-025-00720-0, published online 09 May 2025

The original version of this Article contained errors in the captions of Figures 1, 2 4, 5 and 6;

The caption of Figure 1 was incorrectly given as:

An example of a local file-based storage system for HCS bioimaging. After image acquisition, data can be transferred to a shared repository. If additional metadata is available (e.g., information on experimental conditions or the biological sample), it can be added and transferred to the image storage location. From this location, data can be fetched to perform further analysis or be shared on public repositories (e.g., Image Data Repository (IDR), BioImage Archive (BIA)). The figure was created using to BioRender.

now reads:

An example of a local file-based storage system for HCS bioimaging. After image acquisition, data can be transferred to a shared repository. If additional metadata is available (e.g., information on experimental conditions or the biological sample), it can be added and transferred to the image storage location. From this location, data can be fetched to perform further analysis or be shared on public repositories (e.g., Image Data Repository (IDR), BioImage Archive (BIA)). The figure was created in BioRender. Massei, R. (2025) https://BioRender.com/g74o795.

The caption of Figure 2 was incorrectly given as:

A potential automated high content screening (HCS) for image data management workflow using a dedicated workflow management system integrated with OMERO. Image was created thanks to BioRender.

now reads:

A potential automated high content screening (HCS) for image data management workflow using a dedicated workflow management system integrated with OMERO. Image was Created in BioRender. Massei, R. (2025) https://BioRender.com/k21s891.

The caption of Figure 4 was incorrectly given as:

Two examples of workflows for OMERO upload using Galaxy and KNIME (Workflow 1). Testing datasets are available on the Broad Bioimage Benchmark Collection under the accession number BBBC014 (Ljosa et al., 2012). Image was created thanks to BioRender.

now reads:

Two examples of workflows for OMERO upload using Galaxy and KNIME (Workflow 1). Testing datasets are available on the Broad Bioimage Benchmark Collection under the accession number BBBC014 (Ljosa et al., 2012). “Image was created in BioRender. Massei, R. (2025) https://BioRender.com/xiy0tjb and https://BioRender.com/A69m173.

The caption of Figure 5 was incorrectly given as:

Overview on Workflow 2 “Zebrafish embryos HCS data analyzed with FishInspector”. Image was created thanks to BioRender.

now reads:

Overview on Workflow 2 “Zebrafish embryos HCS data analyzed with FishInspector”. Image was created Created in BioRender. Massei, R. (2025) https://BioRender.com/wewmfnw.

The caption of Figure 6 was incorrectly given as:

Overview on Workflow 3 “Nuclei segmentation of cell lines in Galaxy”. Image was created thanks to BioRender. A better overview of the workflow in higher resolution can be found at the WorkflowHub under the accession number 1259.

now reads:

Overview on Workflow 3 “Nuclei segmentation of cell lines in Galaxy”. Image was Created in BioRender. Massei, R. (2025) https://BioRender.com/8awy78c. A better overview of the workflow in higher resolution can be found at the WorkflowHub under the accession number 1259.

The original Article has been corrected.

